# Generation of prostate cancer assembloids modeling the patient-specific tumor microenvironment

**DOI:** 10.1371/journal.pgen.1011652

**Published:** 2025-03-31

**Authors:** Juhee Lee, Yunhee Kim, Cheol Lee, Seong Soo Jeon, Hae Seo, Jongwon Lee, Jungmin Choi, Minyong Kang, Eunjee Kim, Kunyoo Shin

**Affiliations:** 1 Institute of Molecular Biology and Genetics, Seoul National University, Seoul, Republic of Korea; 2 School of Biological Sciences, College of Natural Sciences, Seoul National University, Seoul, Republic of Korea; 3 Department of Pathology, Seoul National University College of Medicine, Seoul, Republic of Korea; 4 Department of Urology, Samsung Medical Center, Sungkyunkwan University School of Medicine, Seoul, Republic of Korea; 5 Department of Biomedical Sciences, Korea University College of Medicine, Seoul, Republic of Korea; 6 Department of Health Sciences and Technology, The Samsung Advanced Institute for Health Sciences & Technology (SAIHST), Sungkyunkwan University, Seoul, Republic of Korea; Shanghai Institute of Biochemistry and Cell Biology, Chinese Academy of Sciences, CHINA

## Abstract

Prostate cancer (PC) is the most frequently diagnosed malignancy among men and contributes significantly to cancer-related mortality. While recent advances in *in vitro* PC modeling systems have been made, there remains a lack of robust preclinical models that faithfully recapitulate the genetic and phenotypic characteristics across various PC subtypes—from localized PC (LPC) to castration-resistant PC (CRPC)—along with associated stromal cells. Here, we established human PC assembloids from LPC and CRPC tissues by reconstituting tumor organoids with corresponding cancer-associated fibroblasts (CAFs), thereby incorporating aspects of the tumor microenvironment (TME). Established PC organoids exhibited high concordance in genomic landscape with parental tumors, and the tumor assembloids showed a higher degree of phenotypic similarity to parental tumors compared to tumor organoids without CAFs. PC assembloids displayed increased proliferation and reduced sensitivity to anti-cancer treatments, indicating that PC assembloids are potent tools for understanding PC biology, investigating the interaction between tumor and CAFs, and identifying personalized therapeutic targets.

## Author summary

Prostate cancer is one of the most common cancers worldwide and a leading cause of cancer-related death among men. However, understanding its progression and predicting therapeutic responses remain challenging due to the lack of models that faithfully recapitulate the genetic and phenotypic features of these tumors, particularly since existing models often fail to incorporate the tumor microenvironment. Here, we developed patient-specific prostate cancer assembloids, spanning from low-grade localized to castration-resistant prostate cancers, by three-dimensionally reconstituting patient-derived tumor organoids with cancer-associated fibroblasts. We found that these prostate cancer assembloids mimic key pathophysiological characteristics of the parental tumors, including the tissue architecture, tumor growth, and responses to anti-cancer treatments. Our findings suggest that prostate cancer assembloids can serve as a powerful platform not only for studying prostate cancer biology but also for developing new drugs and personalized therapeutic options.

## Introduction

Prostate cancer (PC) is the most prevalent cancer among men worldwide and a leading contributor to cancer-related mortality [1]. The initial diagnosis of most PC patients reveals localized hormone-naïve PC; however, over time, a subset of individuals develops castration-resistant PC (CRPC), a state associated with a poor prognosis. The diversity of histological, molecular, and genomic traits among patients, and even within individual tumors, presents diagnostic hurdles and impedes the implementation of precision medicine [2–8]. These findings emphasize the need for reliable preclinical models to understand the tumor dynamics and heterogeneity of PC.

Recently, advancements of *in vitro* culture techniques have led to the development of models known as organoids, facilitating the study of various human diseases, including cancers [9–11]. These models have been established for PC, encompassing a range of phenotypes and subtypes [12], such as metastatic PC [13,14] and small cell neuroendocrine PC [15]. However, few studies focus on organoids derived from localized PC (LPC), especially those capable of accurately replicating the genomic and molecular heterogeneity of primary sites [16].

Cancer-associated fibroblasts (CAFs) are pivotal components of the tumor microenvironment (TME), playing a central role in cancer development through either direct interaction with cancer cells [17] or cytokine release [18–21]. CAFs significantly contribute to carcinogenesis, tumor progression [22–24], and the development of resistance to anti-cancer therapeutics [25]. However, conventional PC organoids, which are composed solely of tumor epithelial cells, fail to precisely model the *in vivo* interactions between tumor cells and CAFs within the TME.

To overcome the limitations of existing models, we aimed to develop patient-specific *in vitro* cancer models by establishing organoids derived from PC specimens. These tumor organoids retained key phenotypic features and closely reflected the genetic alterations of the parental tumor tissues. To further enhance the physiological relevance of our model to PC, we created PC assembloids by reconstituting PC organoids with matched CAFs [26]. Compared with organoids lacking CAFs, PC assembloids faithfully mimicked the morphological characteristics of parental tumor tissues. We showed that the presence of CAFs in assembloids led to enhanced proliferation of PC cells and reduced sensitivity to anti-cancer treatments. Thus, our study indicates the potential of a patient-specific tumor assembloid model for studying the dynamic interactions between cancer cells and CAFs as well as tumor dynamics and heterogeneity of PC.

## Results

### Establishment of patient-specific PC organoids

**Fig 1:**
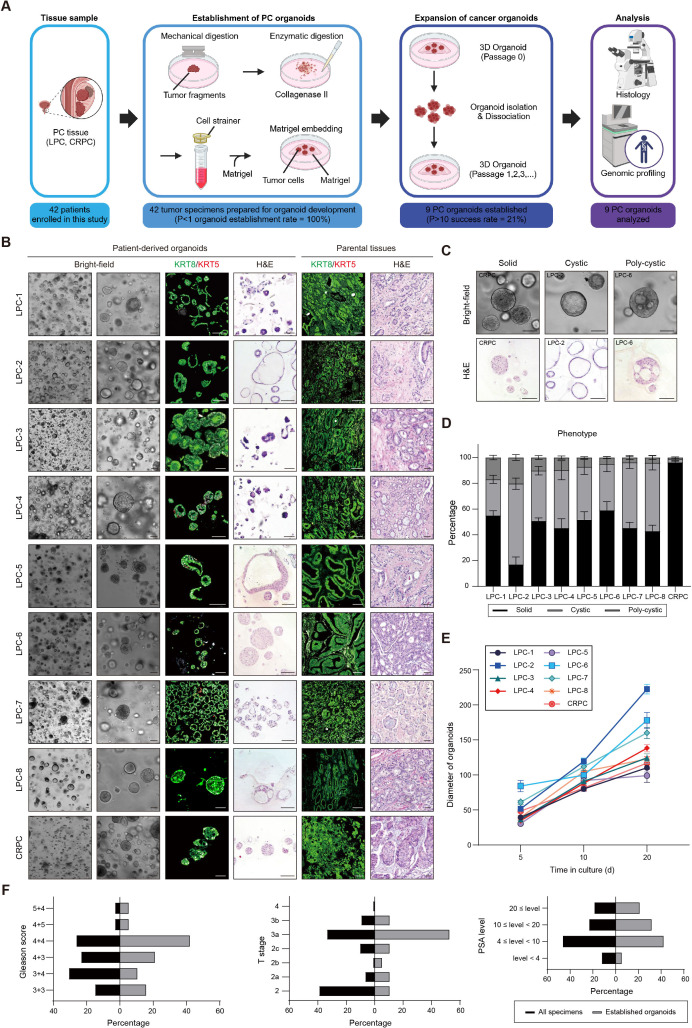
Establishment of patient-specific PC organoids from LPC and CRPC specimens. (A) Schematic diagram illustrating the establishment of patient-derived PC organoids. Created in BioRender. Kim, Y. (2025) https://BioRender.com/d84u314. (B) Establishment of nine PC organoids from patients with LPC and CRPC. The histopathology of PC organoids and parental tissues was analyzed using haematoxylin and eosin (H&E) staining and immunostaining for luminal (KRT8) and basal (KRT5) markers. Scale bars = 100 μm. (C) Bright-field and H&E-stained images showing the morphological phenotype of PC organoids. (D) Quantification of the proportion of each organoid morphology at day 20 of culture. (E) Quantification of the average size of organoids at 5, 10, and 20 days. (F) Bar chart illustrating the stratification of all tumors and all derived organoid lines based on Gleason score (left), T stage (middle), and PSA level (ng/mL) (right). Gleason scores, T stage, and PSA levels were determined from the parental tumor tissues.

In this study, we successfully generated nine distinct organoid lines from PC tissue samples obtained from nine patients who underwent prostatectomy, with informed consent obtained (Table 1). We isolated tumor cells through a combination of mechanical disruption and enzymatic digestion, then embedded them in Matrigel matrix and cultured them in an optimized PC organoid culture medium (Fig 1A) [13,14]. Among these organoid lines, eight (LPC-1 through LPC-8) were established from LPC tissues, while the ninth (CRPC) was derived from CRPC tissue localized to the prostate gland (Fig 1B).

**Table 1:** Summary of patient-derived PC organoid lines and the corresponding clinical data.

No.	Disease status	Type of sample	Sampling site	PSA at sampling	Bx_Gleason score	T stage	N stage	M stage	Metastatic sites
LPC-1	Localized PC (Hormone-naïve)	Surgical specimen	Prostate	9.75	4+3	3a	0	0	0
LPC-2	Localized PC (Hormone-naïve)	Surgical specimen	Prostate	6.64	4+4	3a	0	0	0
LPC-3	Localized PC (Hormone-naïve)	Surgical specimen	Prostate	4.46	3+4	3a	0	0	0
LPC-4	Localized PC (Hormone-naïve)	Surgical specimen	Prostate	10.2	3+3	2a	0	0	0
LPC-5	Localized PC (Hormone-naïve)	Surgical specimen	Prostate	8.39	3+3	2c	0	0	0
LPC-6	Localized PC (Hormone-naïve)	Surgical specimen	Prostate	23.14	4+3	2b	0	0	0
LPC-7	Localized PC (Hormone-naïve)	Surgical specimen	Prostate	21.5	4+3	3a	0	0	0
LPC-8	Localized PC (Hormone-naïve)	Surgical specimen	Prostate	7.66	3+4	2	0	0	0
CRPC	mCRPC	TUR chip	Prostate	26.6	4+3	4	1	1	LNs, bones

LPC = localized prostate cancer; CRPC = castration-resistant prostate cancer; mCRPC = metastatic castration-resistant prostate cancer; TUR = transurethral resection; PSA = prostate-specific antigen; Bx = biopsy; LN = lymph node.

PC organoids exhibited morphological features similar to those of patient tumor tissues (Fig 1B). PC organoids maintained their acinar or solid structures and displayed a triad of morphologies—solid, cystic, and poly-cystic (Fig 1B–D)—within a one-week span post embedding. While tumor organoids derived from LPC showed various morphologies, those derived from CRPC tumor tissue primarily formed solid structures (Fig 1D). Given that PC typically exhibits slower growth compared to other cancer types, the individual PC organoid lines also displayed a relatively slower growth rate than other types of tumor organoids (Fig 1E) [27–33]. On average, it took approximately 20 days for these tumor organoids to reach a diameter of 100–200 μm (Fig 1E). Based on the Gleason score, T stage, and prostate-specific antigen (PSA) level, we observed no discernible bias in organoid establishment when comparing the stratification of all tumor tissue samples with that of established patient-derived PC organoid lines (Fig 1F).

To further characterize the PC organoid cultures, immunohistochemical analyses were performed using the luminal cell marker keratin 8 (KRT8) and the basal cell marker keratin 5 (KRT5). During the process of tumor organoid establishment from primary PC specimens, previous studies encountered challenges related to the overgrowth of benign epithelial cells as well as the presence of significant basal cells [13,34]. In comparison, the established PC organoids exhibited a luminal phenotype with the absence of basal cells (Fig 1B).

### PC organoids represent genomic profiles of the parental tumor tissues

**Fig 2:**
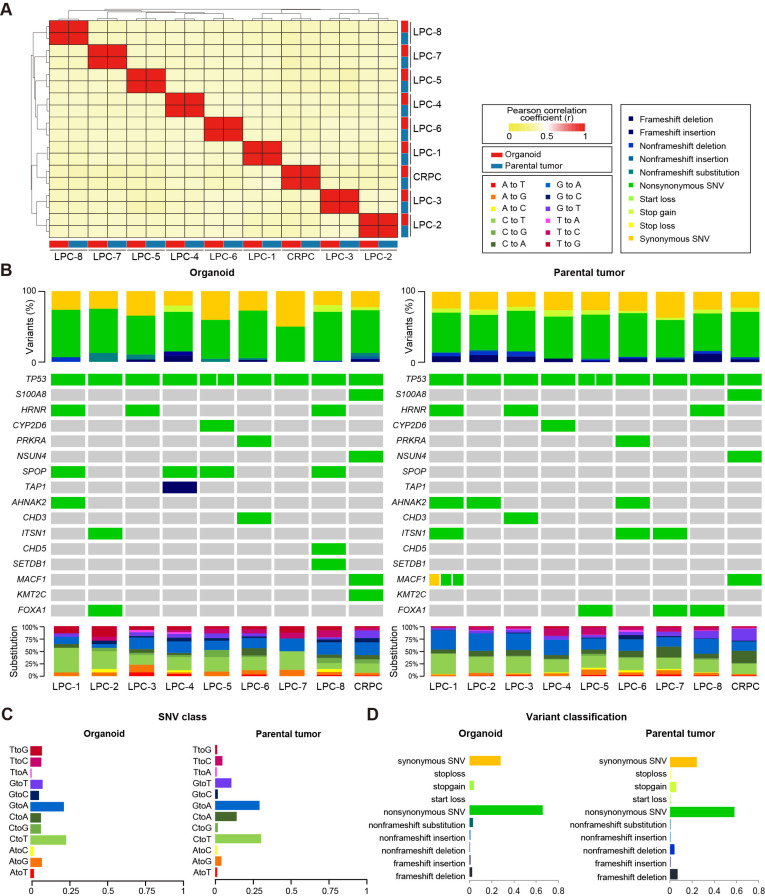
PC organoids represent genomic profiles of the parental tumor tissues. (A) Correlation heatmap of pairwise genotypic concordance among samples, calculated as the Pearson's correlation coefficient (r) based on allele frequency data within the coding regions. (B) Distribution of somatic mutations and oncoplots depicting somatic variants in tumor organoids and parental tissues. (C) Proportion of nucleotide substitution types among total SNVs in organoids and corresponding parental tissues. (D) Frequency of somatic variants in tumor organoids and corresponding parental tissues. Sample annotations are indicated on the right side of panel A. SNV = single-nucleotide variant.

We performed whole-exome sequencing (WES) on the nine established PC organoids to analyze their genomic landscape along with their corresponding tumor tissues. The mean coverage of the WES was 151.9 for tumor tissues and 182.0 for PC organoids. To evaluate the genomic fidelity of the PC organoids, we analyzed genotypic concordance and somatic variant profiles. A high degree of genotypic concordance was observed within the coding regions of the PC organoids compared to their parental tissues (Fig 2A). This high degree of patient-specific similarity indicates that the tumor organoids were successfully derived and established from the parental tumor tissues.

We further analyzed somatic variants based on a set of commonly mutated genes associated with PC, as identified in previous studies [35–37]. We found that tumor organoids recapitulate several key mutational features of PC (Fig 2B–D and S1 Table). All organoids shared the *TP53* mutation with their corresponding parental tissues (Figs 2B and S2A Fig). In addition, several somatic mutations found in patient tumors, such as *S100A8*, *HRNR*, *PRKRA*, and *NSUN4* [35,38–41], were conserved in the corresponding PC organoids (Fig 2B). Moreover, we observed mutations in genes frequently associated with PC, such as *SPOP*, *FOXA1*, and *KMT2C*, in the tumor organoids [35,42,43] (S2B Fig and S2C Fig).

Furthermore, base substitution patterns and variant classification observed in PC tissues were largely preserved in their corresponding PC organoids (Fig 2C and 2D). The most common and least common base substitutions in PC tissues and organoids were C>T transition and T>A, respectively, consistent with the findings of previous studies on a large cohort of PC samples [44].

### Generation of PC assembloids by three-dimensionally reconstituting tumor organoids with matched CAFs

**Fig 3:**
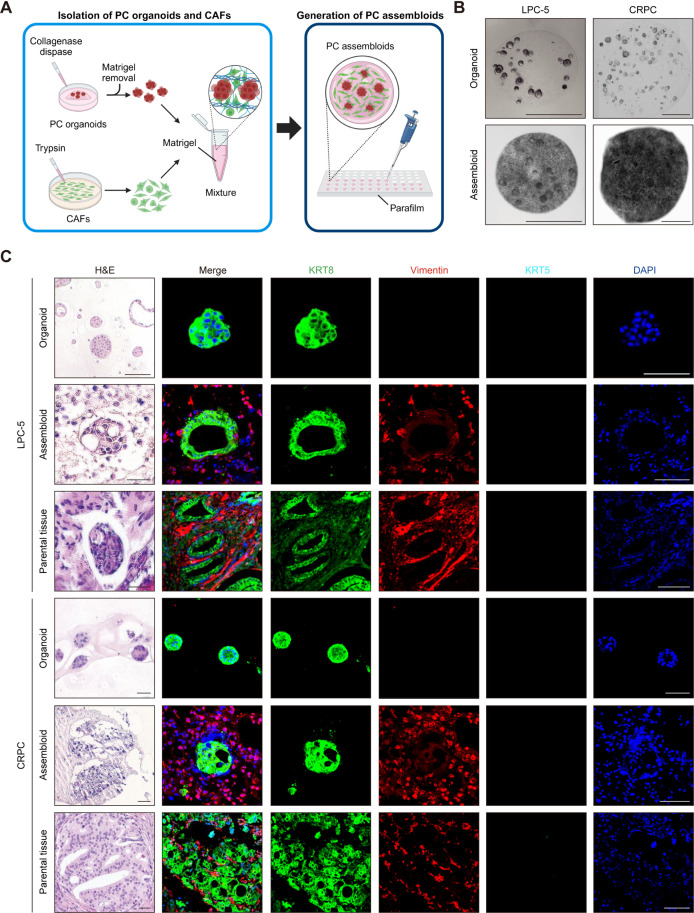
Generation of patient-specific PC assembloids by three-dimensionally reconstituting tumor organoids with matched CAFs. (A) Schematic diagram illustrating the generation of PC assembloids reconstituted with tumor organoids and CAFs. Created in BioRender. Kim, Y. (2025) https://BioRender.com/k74v593. (B) Representative bright-field images of PC organoids and assembloids derived from LPC-5 and CRPC. Scale bar = 1 mm. (C) Histopathology of PC organoids, assembloids, and parental tumor tissues derived from patients with LPC and CRPC: LPC-5 and CRPC analyzed by H&E staining and immunostaining to mark luminal (KRT8) cells, basal (KRT5) cells, and CAFs (vimentin). Scale bar = 100 μm.

To overcome the limitations of traditional organoid models, which only represent the cellular composition and architecture of *in vivo* tumor cells without surrounding tissue stroma or microenvironments, we generated *in vitro* PC assembloids by three-dimensionally reconstituting two representative PC organoids—one derived from LPC (LPC-5) and the other from CRPC—with matched CAFs derived from the patients [26] (Fig 3A). To this end, CAFs were separately cultured from the same tumor samples (S1 Fig). CAFs displayed an elongated and spindle-like morphology and were positive for vimentin and negative for pan-keratin, with variable expression of α-smooth muscle actin (α-SMA) (S1 Fig).

We observed changes in the morphological patterns of tumor growth in reconstituted LPC and CRPC assembloids cultured for 10 days (Fig 3B), which displayed increased phenotypic similarity to parental tumor tissues compared to tumor organoids cultured alone (Fig 3C). KRT8-positive tumors in PC assembloids derived from LPC-5 and CRPC grew in multiple lumps, while being separated by the surrounding CAFs (Fig 3C). These PC assembloids mimicked the histopathology of parental tumors in that KRT8-positive tumors formed nests or clusters ranging from small to large in size, with a clear border distinguished from the stromal compartment (Fig 3C). Of note, tumor cells within both the LPC and CRPC assembloids retained luminal subtypes consistent with their parental tumor tissues (Fig 3C), all of which suggest that PC assembloids faithfully recapitulate the intrinsic tumor architecture and histopathology of the parental tumors, which are often not preserved in conventional tumor organoids.

### CAFs promote tumor proliferation and attenuate drug sensitivity in PC assembloids

**Fig 4:**
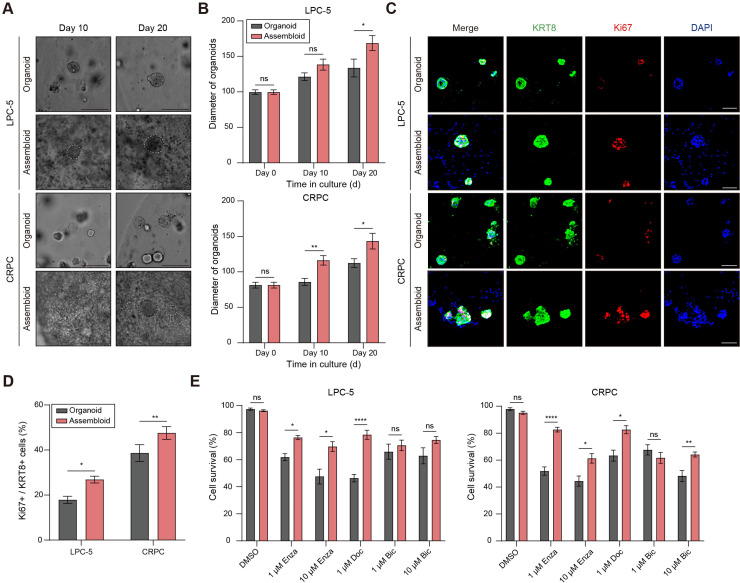
CAFs promote tumor proliferation and attenuate drug sensitivity in PC assembloids. (A) Representative bright-field images of PC organoids and assembloids derived from LPC-5 and CRPC. Dotted lines demarcate the border between organoids and CAFs. Scale bars = 500 μm. (B) Graphs illustrating the average size of tumors at 0, 10, and 20 days. (C) Immunofluorescence images of PC organoids and assembloids stained with KRT8, Ki67, and DAPI. Scale bars = 200 μm. (D) Graph showing the proportion of Ki67-positive tumor cells in KRT8-positive PC cells of PC organoids or assembloids (*n* = 6). (E) Graphs depicting the proportion of cleaved caspase-3-positive tumor cells in PC organoids and assembloids treated with enzalutamide (1 and 10 μM), bicalutamide (1 and 10 μM), docetaxel (1 μM), and vehicle control for 72 h (*n* = 6). Scale bars = 100 μm. Data are mean +/− SEM. n.s., not significant; * p-value < 0.05, ** p-value < 0.01, and **** p-value < 0.0001 as determined by unpaired *t*-test. DMSO = dimethyl sulfoxide; Bic = bicalutamide; Enz = enzalutamide; Doc = docetaxel.

To examine the influence of CAFs on the proliferation of tumor organoids, we measured the size of the PC organoids within the assembloids. Notably, the average diameter of the PC organoids in both the LPC and CRPC assembloids increased significantly (Fig 4A and 4B). At the onset of assembloid culture, the average diameter of the PC organoids ranged between 80–100 µm, but after 20 days of culture with CAFs, this diameter expanded by more than 25 µm, compared to that of the organoids cultured alone (Fig 4A and 4B).

Furthermore, we analyzed the expression of the proliferation marker Ki67 via immunohistochemical analysis. Interestingly, we identified a substantial increase in Ki67-positive cells among KRT8-positive cells in PC assembloids, relative to PC organoids in both LPC and CRPC assembloids (Fig 4C and 4D). In addition, compared to conventional co-culture systems, PC assembloids demonstrated enhanced tumor growth, as evidenced by larger tumor size and increased Ki67 expression (S3 Fig). Ki67 expression in CAFs was rarely detected, suggesting minimal proliferative activity of CAFs within the three-dimensional assembloid model. Overall, CAFs promote the proliferation of tumor organoids in both the LPC and CRPC, consistent with previous research on the synergistic interactions that promote PC proliferation [20,45,46].

Subsequently, to evaluate the impact of CAFs on conventional therapeutic responses of PC, we exposed PC assembloids and organoids to enzalutamide, bicalutamide, and docetaxel for 72 hours. Therapeutic response was assessed by quantifying the number of cleaved caspase-3-positive cells among KRT8-positive tumor cells, which allows the evaluation of drug-induced tumor cell apoptosis. Both PC organoids and assembloids exhibited a significant decrease in tumor growth in response to enzalutamide, docetaxel, and bicalutamide (Fig 4E and S4 Fig). However, assembloids of LPC (LPC-5) showed decreased drug responses to enzalutamide (1 and 10 μM) and docetaxel (1 μM), but not to bicalutamide (1 and 10 μM), compared to organoids (Fig 4E and S4A Fig). Moreover, CRPC assembloids showed reduced drug responses to enzalutamide (1 and 10 μM), docetaxel (1 μM), and bicalutamide (10 μM) compared to organoids alone (Fig 4E and S4B Fig).

Taken together, our findings showed that CAFs play a protective role against tumor cytotoxicity in response to enzalutamide, docetaxel, and bicalutamide, thereby suggesting their involvement in promoting drug resistance in PC. These results align with several studies showing that CAFs act as barriers to anti-cancer drugs, hampering effective treatment in solid tumor tissues [47,48].

## Discussion

Despite significant advances in the treatment of patients with PC, patient responses to various therapeutic regimens remain heterogeneous. The lack of patient-specific PC models that precisely mimic the complex tumor-stroma interactions *in vivo,* hinders the advancement of personalized treatment strategies and PC research. Here, we successfully established organoids derived from patients with LPC and CRPC that retained key histological characteristics and genetic heterogeneity observed in the parental tumor tissues. By reconstituting patient-matched CAFs with tumor organoids, we generated PC assembloids that mimic the histopathology and drug responses of PC tissues, leading to a better understanding of the dynamic cellular interactions between cancer cells and CAFs in PC.

Numerous studies have contributed to the development of PC organoid models, underscoring their utility in capturing PC heterogeneity and modeling drug responses. Gao et al. (2014) generated organoids from metastatic lesions and circulating tumor cells of patients with metastatic CPRC. These organoids faithfully retained the histological and molecular characteristics of their parental tumors, recapitulated the heterogeneity of CRPC, and demonstrated their applicability for *in vitro* drug testing [13,14]. Similarly, Puca et al. (2018) established organoids from metastatic neuroendocrine PC, showing genomic, transcriptomic, and epigenomic concordance with patient tumors [15]. However, neither study achieved the successful establishment of tumor organoids from primary PC. Recently, two independent studies reported the development of PC organoids from treatment-naïve PC patients, demonstrating that these organoids recapitulate the histopathological features of parental tumors and exhibit distinct drug responses across samples [16,34]. Nonetheless, these studies were limited by their inability to maintain the pathophysiological characteristics observed *in vivo* within organoids during extended culture periods.

In this study, we successfully established eight tumor organoids derived from patients with LPC, particularly those with low-grade tumors, as well as one organoid derived from a CRPC patient. These organoids not only recapitulated histological and luminal features of PC tissues but also exhibited key genomic alterations, including mutations in *TP53* and *HRNR* (Fig 2B). The successful establishment of tumor organoids directly from LPC—a tumor type characterized by inherently low proliferation rates—marks a significant advancement, particularly as it eliminates the need for xenograft models, such as patient-derived xenografts [16,49]. This achievement provides a robust platform for studying primary LPC within a controlled *in vitro* environment, thereby facilitating more precise modeling of disease progression and therapeutic responses.

Another significant advance of our study is the development of patient-derived PC assembloids by reconstituting PC organoids with patient-matched CAFs. To our knowledge, the reconstitution of human LPC organoids or CRPC organoids with matched CAFs derived from the same cancer region has not been previously reported. Compared to conventional PC organoids without CAFs, PC assembloids more precisely recapitulated the pathophysiological features of parental tumor tissues, including tissue architecture and tumor growth dynamics. The incorporation of CAFs in PC assembloids not only increased the tumor growth but also reduced the sensitivity to various therapeutic drugs, such as enzalutamide, bicalutamide, and docetaxel. These findings suggest that CAFs may function as physical barriers to cancer cells, which is consistent with prior research indicating that CAFs produce a protective shield around cancer cells by remodeling the extracellular matrix [50] and secreting factors such as WNT16B [51] that mediate resistance to chemotherapy and targeted therapies. Thus, our patient-derived, three-dimensional PC assembloids may offer a promising platform for investigating the interactions between cancer cells and CAFs within the TME and predicting patient-specific drug responses *in vitro* within a clinical context.

Lastly, our assembloid model system has significant potential for a range of applications, allowing genetic manipulations to be applied independently to various cell types in a combinatorial manner. Emerging evidence has suggested that dynamic interactions of tumors with tumor stroma are critical for cancers to develop therapy resistance. Genetically manipulated tumor assembloids, in which genetic modifications of several candidate genes are applied independently to both tumor organoids and CAFs in a combinatorial manner, can provide insight into the role of CAFs in tumor progression, lineage plasticity from LPC to CRPC or neuroendocrine PC, and drug resistance. Moreover, these genetically manipulated assembloids can be used to investigate potential therapeutic strategies targeting the crosstalk between CAFs and cancer cells, potentially leading to novel approaches for cancer treatment.

Taken together, our study, which has the potential to profoundly enhance our understanding of the role of complex crosstalk between cancer cells and CAFs in tumor progression and drug resistance, will facilitate the development of an innovative model platform for studying various types of human cancers and serve as a valuable tool for evaluating the efficacy of anti-cancer drugs and exploring novel therapeutic approaches in a patient-specific manner.

## Materials and methods

### Ethics statement

Human PC tissues were obtained from the Samsung Medical Center (SMC). Specimens (0.5–1 cm^3^) were obtained from individuals who underwent either radical prostatectomy or transurethral resection of the prostate (Table 1) according to protocols approved by the Institutional Review Board of SMC (IRB number: SMC 2019-03-064). Informed formal written consent was obtained from all participants, and all experiments using human samples were performed following IRB guidelines. The fresh radical prostatectomy tumor specimens were examined by expert pathologists.

### PC organoid culture

To establish organoids from human PC tissues, the tissues were minced and incubated in Dulbecco’s modified Eagle’s medium (DMEM) containing 10% fetal bovine serum (FBS), 1% penicillin/streptomycin, 10 μM Y-27632 (Abmole), 10 mM HEPES (pH 7.4, Sigma), and 2 mg/mL collagenase type II (Thermo Fisher Scientific) for 1 h at 37 °C with trituration every 30 min. After digestion, the suspension was filtered through a 100 μm cell strainer. The dissociated clusters were centrifuged and resuspended in ammonium-chloride-potassium (ACK) lysing buffer (Invitrogen) to remove red blood cells. The cells were counted, and the tumor cells were resuspended in Matrigel (growth factor reduced phenol red-free; Corning) (40 μL of 100% Matrigel per 50,000 cells). The mixture was placed in the center of a well in a 24-well tissue culture plate and solidified for 20 min at 37 °C. Pre-warmed organoid culture medium (indicated in Table 2) was added. For the first day, 10 μM Y-27632 was added to the medium. The medium was changed every 2–3 days and passaged the organoids at a 1:2–3 ratio every 2–4 weeks. For passaging, tumor organoids embedded in Matrigel were released by physical pipetting, collected, and centrifuged at 1,500 rpm for 5 min at 4 °C. The organoids were then dissociated into single cells by incubating them in 0.25% trypsin-EDTA (Welgene) supplemented with 10 μM Y-27632 for 3 min at 37 °C, followed by 1–2 min of trituration. The cells were seeded in Matrigel and cultured, as described above.

**Table 2:** Composition of PC organoid culture medium.

Reagent	Stock concentration	Final concentration
Advanced DMEM/F12	–	–
HEPES	1 M	10 mM
GlutaMAX	100X	1X
Nicotinamide	1 M	10 mM
Penicillin/streptomycin	100X	1X
NAC	200 mM	1.25 mM
Primocin	50 mg/mL	100 μg/mL
B27	50X	1X
EGF	100 μg/mL	50 ng/mL
A83-01	25 mM	500 nM
FGF10	10 μg/mL	10 ng/mL
FGF2	10 μg/mL	1 ng/mL
SB202190	30 mM	10 μM
PGE2	10 mM	1 μM
DHT	10 μM	1 nM (for LPC) or 0.1 nM (for CRPC)
Noggin	100 μg/mL	100 ng/mL
R-spondin-1	–	R-spondin-1 10% conditioned medium

LPC = localized prostate cancer; CRPC = castration-resistant prostate cancer; NAC= N-acetylcysteine; EGF= epidermal growth factor; DHT= dihydrotestosterone; FGF2= basic fibroblast growth factor; FGF10= fibroblast growth factor 10; PGE2= Prostaglandin E2.

### Isolation and culture of CAFs from human PC specimens

Patient-derived CAFs were isolated and cultured as follows. During the culture period of PC organoids, CAFs proliferated as adherent cells on the bottom of the culture plates, distinct from the PC organoids embedded in Matrigel droplets. To isolate these CAFs, the Matrigel droplets containing PC organoids were manually removed using a pipette. The culture plates were then washed with phosphate-buffered saline (PBS) once to remove any remaining Matrigel or non-adherent cells. The adherent cells remaining on the plate were then detached by treating them with 0.25% trypsin-EDTA for 2–3 min at 37 °C. The detached cells were then replated onto culture plates in CAF culture medium [Medium 106 (Gibco) supplemented with Low Serum Growth Supplement (Gibco)]. The medium was changed every 2–3 days to maintain optimal growth conditions. CAFs were subcultured at a 1:2 ratio upon reaching 80% confluence. To minimize potential contamination from cancer cells, CAF cultures were passaged at least three times, and immunocytochemistry was performed using antibodies against pan-keratin, vimentin, and α-SMA to confirm their identity as fibroblasts. CAFs between passages 5 and 10 were used for experimental studies.

### Generation of three-dimensional human PC assembloids

To create human PC assembloids, we reconstituted PC organoids and CAFs, following the protocol as described in [26]. PC organoids were cultured for 10 days, reaching a diameter of approximately 60–100 μm, while CAFs were grown separately in two-dimensional culture until they reached 90% confluence. To determine the patient-specific CAF-to-tumor cell ratio for assembloid generation, immunostaining was performed on sections of the original tumor tissue. Tumor cells and CAFs were identified using antibodies against pan-keratin and vimentin, respectively.

CAFs were detached using 0.25% trypsin-EDTA for 2–3 min at 37 °C, washed with PBS, and resuspended in Matrigel at a density of 2.5 × 10⁵ cells/mL. PC organoids were treated with 0.5 mg/mL collagenase/dispase solution for 1 h at 37 °C, followed by washing, centrifugation, and then resuspension of the collected organoids in 1 mL of Matrigel containing CAFs at a pre-determined concentration matching the CAF-to-tumor cell ratio. Prior to resuspending the tumor organoids in Matrigel, approximately 5% of the organoid droplets were dissociated into single cells for counting to estimate the number of tumor cells within the organoids. A 4 μL mixture of organoids and CAFs was placed on a sheet of parafilm to make droplets. The droplets were then flipped, solidified for 15 min at 37 °C, and transferred to a 60-mm petri dish containing 5 mL of assembloid medium (50% organoid culture medium and 50% CAF culture medium). The plates were incubated in a shaking incubator (Eppendorf) rotating at 80 rpm at 37 °C and 5% CO_2_. The medium was changed every 2 days until analysis.

Assembloids were used for experimental studies after 10 or 20 days of culture. For controls, PC organoids were embedded in Matrigel without CAFs, following the same protocol.

### Histological analysis

Tissue specimens were prefixed in 10% neutral-buffered formalin for 24 h and embedded them in paraffin. The organoids were embedded in 2% agarose gel to create agarose blocks, which were fixed in 10% neutral-buffered formalin for 24 h and embedded in paraffin. Paraffin blocks were sectioned into 4-μm sections using a microtome and stained the slides with H&E for histological analysis.

### Immunohistochemistry and immunocytochemistry

For immunostaining of frozen samples, we fixed tissues, PC organoids, and assembloids constituted with tumor organoids and CAFs in 4% paraformaldehyde (PFA)-PBS for 15 min at 4 °C and cryopreserved them in 30% sucrose-PBS overnight. The samples were embedded in an OCT compound (Sakura) and frozen at -20 °C. Then, we cut them into 10–20-µm sections using a cryostat (Leica). The sections were fixed in 4% PFA-PBS for 15 min at 4 °C, washed with PBS three times, and blocked in 2% goat serum and PBS containing 0.25% Triton X-100 (PBS-T) for 1 h at RT. The sections were incubated overnight at 4 °C in a humidified chamber with the following primary antibodies diluted in a blocking buffer: rabbit anti-keratin 5 (KRT5; ab53121, 1:500, Abcam); rat anti-keratin 8 (KRT8; TROMA-I-c, 1:500, DSHB); mouse anti-pan-keratin (pan-K; 4545S, 1:400, Cell Signaling); chicken anti-vimentin (ab5733, 1:500, Millipore); mouse anti-α-SMA (ab7817, 1:200, Abcam); rabbit anti-Ki67 (ab15580, 1:500, Abcam); rabbit anti-cleaved caspase 3 (9661S, 1:200, Cell Signaling) [52]. The sections were then washed three times with 0.25% PBS-T. We then washed the sections three times with 0.25% PBS-T and incubated them with secondary antibodies (1:1,000, Life Technologies) diluted in blocking buffer together with DAPI for 1 h at RT. Sections were washed twice with 0.25% PBS-T and mounted with Prolong Gold mounting reagent (Invitrogen). Images were acquired with a Nikon AX/AX R confocal microscope system.

For immunostaining of paraffin sections, paraffin-embedded tissue sections were rehydrated using pH-6 sodium citrate buffer prior to antigen retrieval. Following heat-induced antigen retrieval, the sections were washed two times with PBS and then blocked them in blocking buffer for 1 h at RT. The sections were incubated overnight at 4 °C in a humidified chamber with primary antibodies diluted in blocking buffer. After three washes with 0.25% PBS-T, the sections were incubated with secondary antibodies, as appropriate, diluted 1:1,000 in 0.25% PBS-T together with DAPI for 1 h at RT. The sections were washed twice with 0.25% PBS-T and mounted them on slides with Prolong Gold mounting reagent.

For immunocytochemistry, cells were plated on poly-L-ornithine and 5 μg/mL laminin-coated coverslips in a 12-well plate. When the cells reached 80% confluence, they were washed with PBS and fixed in 4% PFA for 5 min at RT. The cells were washed with PBS three times and blocked for 1 h at RT. The cells were then incubated with diluted primary antibodies for 1 h at RT and washed three times with 0.25% PBS-T. The cells were then incubated with secondary antibodies diluted in blocking buffer for 40 min at RT. The cells were washed two times with 0.25% PBS-T and mounted on glass slides.

### Whole-exome sequencing (WES)

Genomic DNA was extracted from each organoid sample (cultured for 100–150 days) using DNeasy Blood & Tissue Kits (QIAGEN), following the manufacturer’s instructions. For formalin-fixed paraffin-embedded tissues, DNA extraction was performed on the pathologist-reviewed sections using the QIAamp DNA FFPE Tissue Kit (QIAGEN), according to the manufacturer’s guidelines.

We analyzed parental tumors (*n* = 9) and tumor organoids (*n* = 9) using SureSelect V5. We used 0.5–2 μg of genomic DNA and ran samples on a HiSeq 2500 instrument in paired-end 200X sequencing depth (Illumina).

### Sequence alignment and somatic variant calling from WES data

Sequencing data were processed following the GATK Best Practices workflow [53,54]. Somatic single nucleotide variants (SNVs) and insertions/deletions (INDELs) were identified in tumor tissues and organoids using GATK4 MuTect2 in tumor-only mode, as matched normal sequencing data were not available.

To effectively eliminate germline variants in these samples, the MuTect2 pipeline incorporated both a germline resource (af-only-gnomad.hg38.vcf.gz) and a panel of normals (1000g_pon.hg38.vcf.gz). Additionally, only variants not reported in the Exome Sequencing Project (ESP) [55] and the 1000 Genomes Project [56], as well as those with a minor allele frequency (MAF) of 0.1% or lower in ExAC [57], gnomAD [58], and TOPMED [59] databases, were retained. To exclude remaining germline mutations, only variants with variant allele frequency (VAF) ≤ 0.3 were regarded as somatic mutations. Somatic mutation oncoplots and genomic concordance were generated and visualized using the R packages [60] Maftools [61] and ComplexHeatmap [62].

### Proliferation and growth analysis of PC organoids

To measure the growth of the PC organoids over time, the organoids were visualized and counted manually under a Nikon ECLIPSE i2-E inverted light microscope. The size and shape of the organoids were assessed using ImageJ. A total of ≥50 organoids/condition/experiment were analyzed to calculate the mean diameter.

### Drug treatment of PC organoids and assembloids

For the therapeutic response, the PC organoids and assembloids were cultured in organoid medium for 10 days in a shaking incubator (Eppendorf) rotating at 80 rpm at 37 °C and 5% CO2 before treating them with anti-cancer drugs. The PC organoids and assembloids were cultured for 72 h in the presence of either enzalutamide (1 and 10 μM; Selleck Chemicals), bicalutamide (1 and 10 μM; MedChemExpress), or docetaxel (1 μM; MedChemExpress). Cell viability was assessed by immunohistochemical analysis using a rabbit anti-cleaved caspase-3 antibody (9661S, 1:400; Cell Signaling) and a rat anti-KRT8 antibody (TROMA-I-c, 1:500; DSHB), followed by nuclear staining with DAPI (1:1,000).

### Statistics

Statistical analysis was carried out using GraphPad Prism ver. 10. All data are presented as mean ± SEM. Comparisons between groups were performed using unpaired *t*-tests. Values of p < 0.05 were considered statistically significant.

## Supporting information

S1 Fig Characterization of patient-matched primary CAFs. Bright-field and immunofluorescence images of CAFs derived from LPC-5 and CRPC. Scale bars = 100 μm. LPC = localized prostate cancer; CRPC = castration-resistant prostate cancer; CAF = cancer-associated fibroblasts; α‐SMA = alpha-smooth muscle actin; Pan-K = pan-keratin.
(TIF)

S2 Fig WES analysis for parental tissues and the matched PC organoids. (A) Lollipop plot of topology of *TP53* alterations in tumor organoids (top) and corresponding parental tumor tissues (bottom). (B) Lollipop plot of topology of *SPOP* alterations in tumor organoids (top) and corresponding parental tumor tissues (bottom). (C) Lollipop plot of topology of *FOXA1* alterations in tumor organoids (top) and corresponding parental tumor tissues (bottom).
(TIF)

S3 Fig Comparative analysis of tumor growth in co-culture systems and assembloids. (A) Representative bright-field images of co-culture systems and tumor assembloids derived from LPC-5 and CRPC models at days 5 and 10. Scale bars = 100 μm. (B) Immunofluorescence analysis of co-culture systems and assembloids, stained for Pan-K, Ki67, and DAPI. Scale bars = 100 μm. (C) Graphs showing the diameter of tumor organoids in LPC-5 and CRPC over 10 days of culture in co-culture systems and assembloids. (D) Graph showing the proportion of Ki67-positive tumor cells derived from LPC-5 and CRPC in co-culture systems and assembloids. CAF = cancer-associated fibroblast; Pan-K = pan-cytokeratin. Data are mean +/− SEM. n.s., not significant; * p-value < 0.05, ** p-value < 0.01, *** p-value < 0.001, and **** p-value < 0.0001 as determined by unpaired *t*-test.
(TIF)

S4 Fig CAFs reduce drug responses in PC assembloids. (A-B) Immunofluorescence images of tumor organoids and tumor assembloids, derived from LPC-5 (A) and CRPC (B), that were treated with enzalutamide (1 and 10 μM), bicalutamide (1 and 10 μM), docetaxel (1 μM), and vehicle control for 72 h, stained with KRT8 and cleaved caspase-3. DAPI staining in blue. Magnified views of the outlined regions in the immunostaining images are shown in the right panels. Scale bars = 100 μm. LPC = localized prostate cancer; CRPC = castration-resistant prostate cancer; DMSO = dimethyl sulfoxide; Bic = bicalutamide; Enza = enzalutamide; Doc = docetaxel; KRT8 = keratin 8.
(TIF)

S1 Table Somatic variants list of tumor organoids and parental tumor tissues. (XLSX)

S1 Data Source data file. (XLSX)
