# Supplementary figures and images for: Generation of prostate cancer assembloids modeling the patient-specific tumor microenvironment
Source: PLoS Genet. 2025 Mar 31;21(3):e1011652. doi: 10.1371/journal.pgen.1011652 (PMC12002641; doi:10.1371/journal.pgen.1011652)

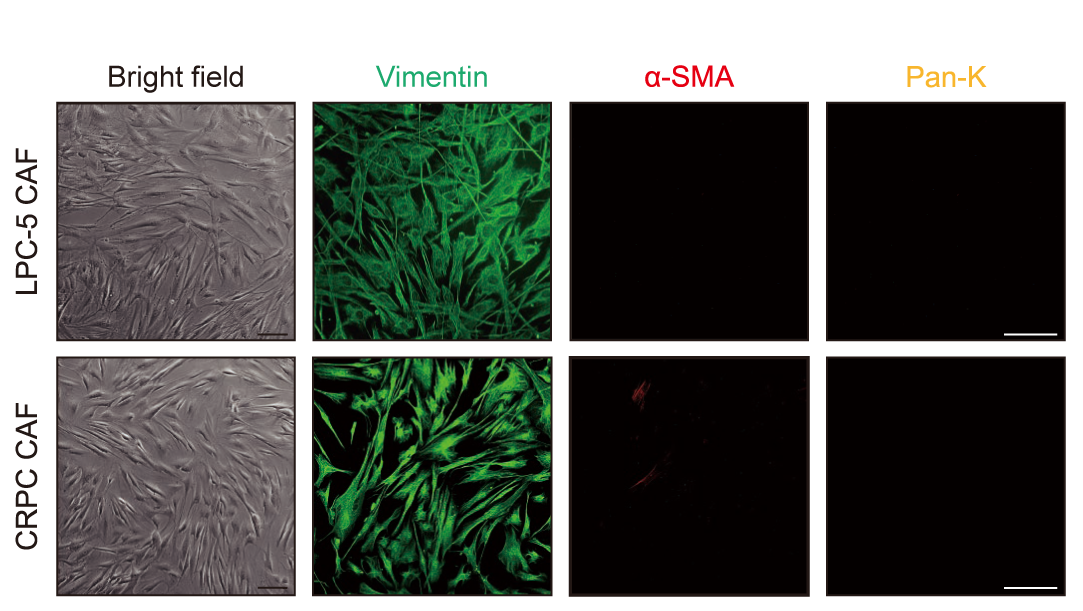

Supplement: S1 Fig — Bright-field and immunofluorescence images of CAFs derived from LPC-5 and CRPC. Scale bars = 100 μm. LPC = localized prostate cancer; CRPC = castration-resistant prostate cancer; CAF = cancer-associated fibroblasts; α‐SMA = alpha-smooth muscle actin; Pan-K = pan-keratin. (TIF) [file pgen.1011652.s001.tif]

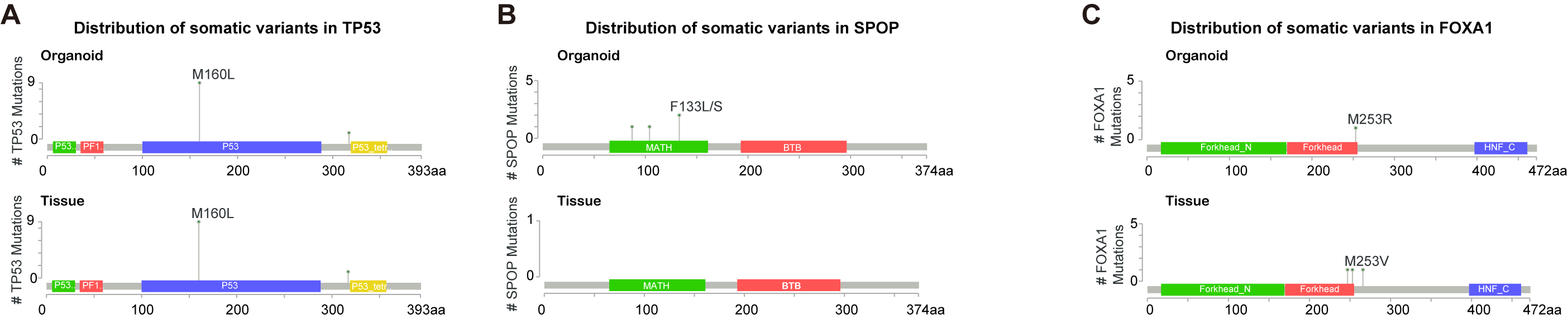

Supplement: S2 Fig — (A) Lollipop plot of topology of TP53 alterations in tumor organoids (top) and corresponding parental tumor tissues (bottom). (B) Lollipop plot of topology of SPOP alterations in tumor organoids (top) and corresponding parental tumor tissues (bottom). (C) Lollipop plot of topology of FOXA1 alterations in tumor organoids (top) and corresponding parental tumor tissues (bottom). (TIF) [file pgen.1011652.s002.tif]

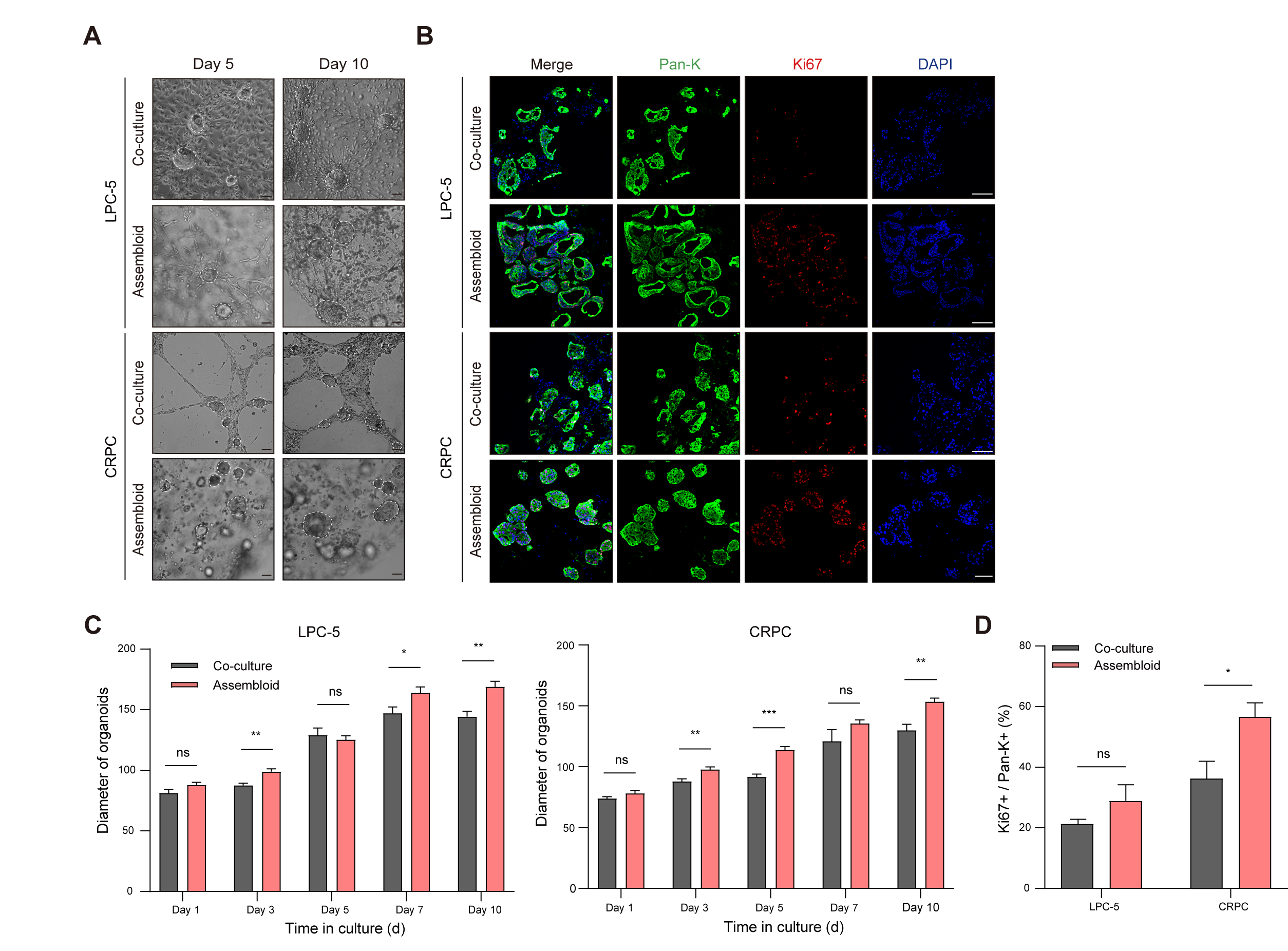

Supplement: S3 Fig — (A) Representative bright-field images of co-culture systems and tumor assembloids derived from LPC-5 and CRPC models at days 5 and 10. Scale bars = 100 μm. (B) Immunofluorescence analysis of co-culture systems and assembloids, stained for Pan-K, Ki67, and DAPI. Scale bars = 100 μm. (C) Graphs showing the diameter of tumor organoids in LPC-5 and CRPC over 10 days of culture in co-culture systems and assembloids. (D) Graph showing the proportion of Ki67-positive tumor cells derived from LPC-5 and CRPC in co-culture systems and assembloids. CAF = cancer-associated fibroblast; Pan-K = pan-cytokeratin. Data are mean +/− SEM. n.s., not significant; * p-value < 0.05, ** p-value < 0.01, *** p-value < 0.001, and **** p-value < 0.0001 as determined by unpaired t-test. (TIF) [file pgen.1011652.s003.tif]

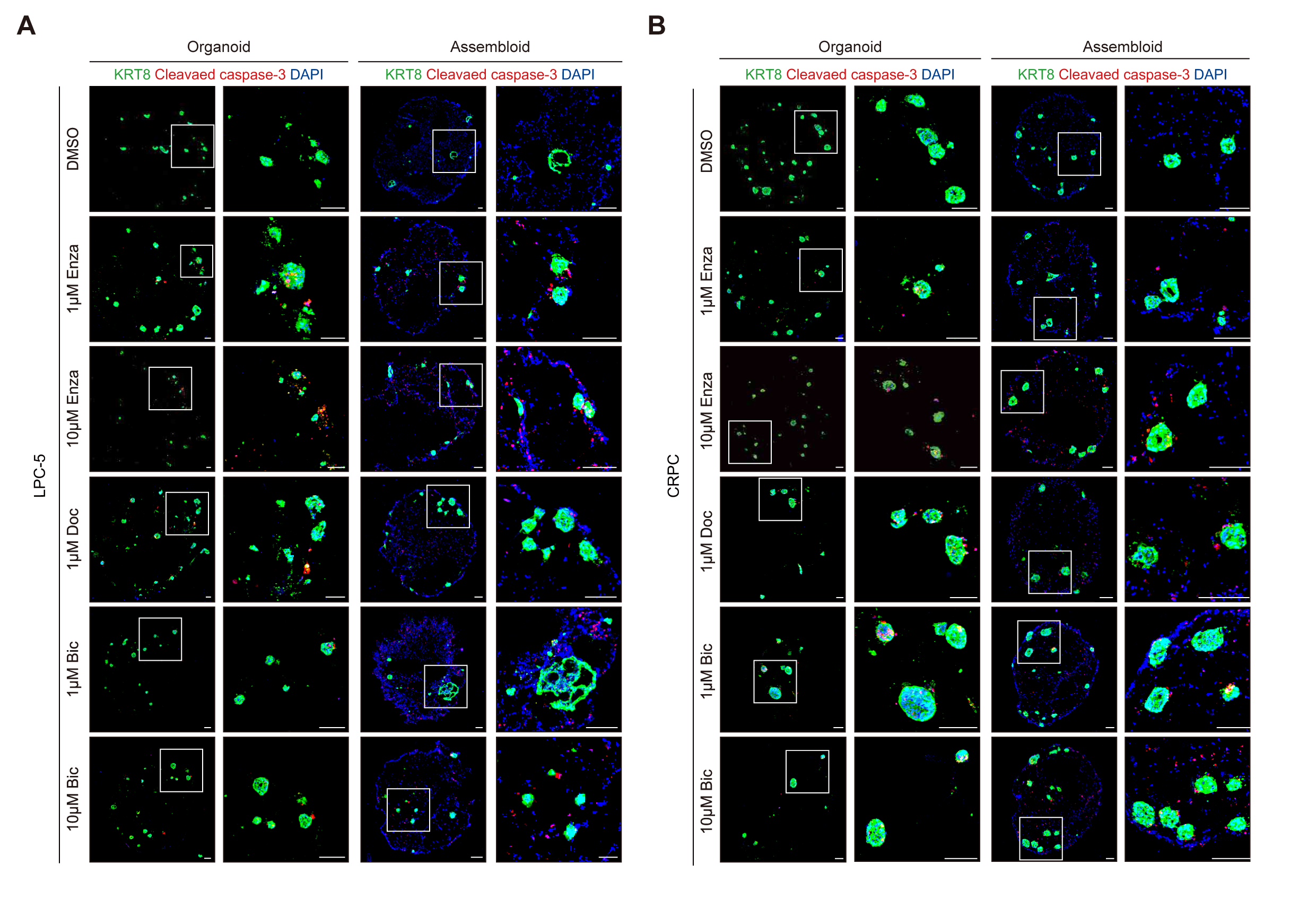

Supplement: S4 Fig — (A-B) Immunofluorescence images of tumor organoids and tumor assembloids, derived from LPC-5 (A) and CRPC (B), that were treated with enzalutamide (1 and 10 μM), bicalutamide (1 and 10 μM), docetaxel (1 μM), and vehicle control for 72 h, stained with KRT8 and cleaved caspase-3. DAPI staining in blue. Magnified views of the outlined regions in the immunostaining images are shown in the right panels. Scale bars = 100 μm. LPC = localized prostate cancer; CRPC = castration-resistant prostate cancer; DMSO = dimethyl sulfoxide; Bic = bicalutamide; Enza = enzalutamide; Doc = docetaxel; KRT8 = keratin 8. (TIF) [file pgen.1011652.s004.tif]
